# System-Wide Change Is Essential to Value the Contributions of Women in Medicine and Science

**DOI:** 10.2196/52509

**Published:** 2023-09-22

**Authors:** Shikha Jain, Jessica M Allan, Rakhee K Bhayani

**Affiliations:** 1 Department of Medicine University of Illinois at Chicago Chicago, IL United States; 2 Palo Alto Medical Foundation Stanford University School of Medicine Palo Alto, CA United States; 3 Division of General Medicine Department of Medicine Washington University School of Medicine St. Louis, MO United States

**Keywords:** women, women physicians, women scientists, gender equity, health care, diversity, leadership, intersectionality, minority tax, gratitude tax, glass ceiling, glass cliff, academia, academic medicine, hierarchy, change

## Abstract

The persistent and pervasive gender gap in health care is a fact backed by data, science, and evidence. This editorial aims to describe some of the challenges that continue to persist. Many of the strategies outlined can be implemented both locally and nationally to effect meaningful change and work toward closing the existing gender gap in health care.

Inequities in our health care systems are pervasive and systemic and have a direct impact on the health care workforce, as well as on patient care delivery and outcomes. Regrettably, due to a lack of female representation in leadership positions, the same women are frequently called upon to address these systemic challenges within our health care systems. To genuinely achieve systemic transformations, we must adopt a comprehensive approach that not only tackles the underlying issues but also avoids burdening the same individuals repeatedly. Our goal in curating and publishing articles from women in medicine and science in this special edition was to collect solution-driven perspectives on the gender gap that continues to permeate our health care systems [1]. We must do more to find intentional solutions for real change.

The persistent invisible work and structural inequities lead to increased rates of burnout for women in medicine [2,3]. During the pandemic, women’s vital yet unseen organizational contributions became apparent. Much of this invisible work focused not only on navigating the challenges that came with a new airborne virus but also on worker well-being, retention, and overall organizational health. Despite being crucial, this work is undervalued, often done by the same few women, eventually leading to burnout [4]. Women experience higher burnout rates than men in part due to structural inequities, and in many cases, this leads to unexpected career shifts or even attrition from the workforce [5]. These types of systemic issues are not unique to the United States; they have been identified in many other countries as well [6]. Additionally, the burden for those with intersectional identities such as women of color is even higher.

For effective and sustained change, we need system-wide structural changes. This essential work must be done by those with a platform and with the ability to make the necessary changes. It must be funded and cannot be relegated to “free time” or “after hours special projects,” lest we continue to perpetuate the uncompensated “third shift” and burn out those women most driven to advocate for change [7]. Individuals in leadership roles must drive structural improvements and allocate resources, avoiding relegation to off-hour projects that perpetuate uncompensated efforts. Additionally, when looking at the big picture, data consistently shows that when there is diversity in leadership, retention and recruitment are better, and when women physicians are taking care of patients, outcomes are better [8,9].

In an effort to counter the biases women in medicine and science face, we created this special edition to highlight solution-driven perspectives.

1. Leaders must address and strategically work to change the lack of female representation in leadership positions within health care. This will require deliberate efforts to promote gender diversity and inclusivity with intentional paths for sponsorship and coaching specifically tailored to support women’s career advancement [10].
2. A transformative shift is needed to recognize and compensate for the invisible work primarily carried out by women in health care. Assigning value and offering protected time for these tasks, including citizenship and leadership roles, is essential. Current relative value unit productivity and academic promotion models inadequately assess the value and time invested in these roles, demanding a reimagining by institutional leadership of reimbursement and promotion strategies in line with the evolving health care workforce.
3. One consistent finding is that women are interested in elevating and sponsoring other women. However, there are a small number of women at the top working hard to lift others as they rise, using a push-pull mentoring strategy [11]. The impact and effectiveness of these strategies have been shown to be successful in different case studies and anecdotally, and can be useful strategies to engage trainees and junior faculty in learning strategies early on that can be used throughout their careers. Senior faculty should invite midcareer faculty to publish with them and then mentor and sponsor them to ensure their success [12].
4. It is essential to dismantle the traditional hierarchical structure in health care that hinders progress toward equity in health care [13]. This would involve re-evaluating existing institutional policies and practices that perpetuate gender bias and adopting more inclusive and flexible models of leadership and decision-making. Allowing junior faculty and trainees to take on leadership roles and sponsoring them for those roles, based on their experience and expertise, can lend itself to effective bidirectional mentorship. By embracing collaborative approaches and empowering individuals at all levels, regardless of gender, we can create a culture of shared responsibility and accountability for driving meaningful change.
5. Another key aspect of achieving systemic change is investing time and money into comprehensive training and education programs promoting inclusive leadership, cultural competence, and diversity awareness [14]. One example is supporting women through women in medicine groups that can provide community building through networking events and professional development through skill-building workshops [15]. Leaders in health care should prioritize investing in these opportunities for their faculty.
6. It is imperative that there are diverse voices and perspectives at the table when working toward shaping health care policies and practices. The work cannot simply be relegated to the individuals who are members of these marginalized groups because this inevitably results in the burnout of those few individuals working toward change. Champions, allies, and accomplices of all genders and races are essential if we are really interested in driving change and moving forward, and they should be invited to participate in discussions addressing the need for change.
7. Creating and fostering networks for growth are key components to the path toward more diverse leadership at the top. Ensuring our networks are as diverse as the communities we serve will allow for more options and chances to approach individuals for opportunities that may otherwise not be considered. Being innovative in creating and fostering these networks is also a key to growing and nurturing these types of networks that often provide the largest opportunities for growth.
8. Social media can be an innovative way to amplify others’ work and an effective way to engage with leaders in the field across specialties. Social media has been found to be effective at helping women advance their careers, allowing for network development and collaborations across institutions and engagement at medical conferences, whether in attendance or via social media [16]. These are innovative approaches to growing networks, amplifying the research and achievements of others, and sponsoring women in medicine that have been found to impact the advancement of women in medicine [17].
9. Those with intersectional identities face double and sometimes triple the burdens and challenges. Organizations must recognize the minority tax and assign real value to diversity, equity, and inclusion work. If we want to see a significant change in our leadership makeup, we must not only sponsor women of color to lead but also set them up for success with support, mentorship, and coaching, and avoid the proverbial “glass cliff.” By actually investing in these efforts, we can increase the probability of advancing exceptional women of color into leadership roles, and strategically, these change agents can innovate and disrupt health care systems for the better [18].
10. Many of these challenges contribute to fueling the burnout epidemic we continue to face within our systems. Addressing burnout in a holistic manner, focusing on prevention by providing resources for coaching, mentorship, or work-life concierge services and by reviewing vacation and family leave and modifying to align with the modern workforce, would go a long way in improving retention of women in the health care workforce [19,20].

Addressing the pervasive inequities in our health care systems requires a multifaceted approach, and these initiatives and strategies must be championed by those in leadership regardless of gender or race. By promoting diversity in health care leadership, valuing and compensating the invisible work performed most often by women, challenging traditional hierarchical structures, investing in training and education, and actively and strategically engaging and sponsoring diverse voices, we have an opportunity to work toward real systemic change. This special edition on women in medicine and science explores unique perspectives and experiences. It also provides actionable strategies to promote a more equitable workplace. We encourage readers to share this collection with key members of their practices and organizations, and shift the responsibilities for change from the few who consistently step up to the rest of the health care community.
